# Mapping Cortical Laminar Structure in the 3D BigBrain

**DOI:** 10.1093/cercor/bhy074

**Published:** 2018-04-18

**Authors:** Konrad Wagstyl, Claude Lepage, Sebastian Bludau, Karl Zilles, Paul C Fletcher, Katrin Amunts, Alan C Evans

**Affiliations:** 1 Department of Psychiatry, University of Cambridge, Cambridge, UK; 2 Department of Neurology and Neurosurgery, Montreal Neurological Institute (MNI), McGill University, Montreal, Canada; 3 Institute of Neuroscience and Medicine (INM-1), Research Centre Jülich, Jülich, Germany; 4 Department of Psychiatry, Psychotherapy and Psychosomatics, RWTH Aachen, Germany; 5 JARA—Translational Brain Medicine, Aachen, Aachen, Germany; 6 Cambridgeshire and Peterborough NHS Foundation Trust, Cambridge, UK; 7 C. and O. Vogt-Institute for Brain Research, University Hospital Düsseldorf, Düsseldorf, Germany

**Keywords:** 3D, Automated layer detection, Cortical folding, Cytoarchitecture, Laminar

## Abstract

Histological sections offer high spatial resolution to examine laminar architecture of the human cerebral cortex; however, they are restricted by being 2D, hence only regions with sufficiently optimal cutting planes can be analyzed. Conversely, noninvasive neuroimaging approaches are whole brain but have relatively low resolution. Consequently, correct 3D cross-cortical patterns of laminar architecture have never been mapped in histological sections. We developed an automated technique to identify and analyze laminar structure within the high-resolution 3D histological BigBrain. We extracted white matter and pial surfaces, from which we derived histologically verified surfaces at the layer I/II boundary and within layer IV. Layer IV depth was strongly predicted by cortical curvature but varied between areas. This fully automated 3D laminar analysis is an important requirement for bridging high-resolution 2D cytoarchitecture and in vivo 3D neuroimaging. It lays the foundation for in-depth, whole-brain analyses of cortical layering.

## Introduction

The isocortex, which forms the major part of the human cerebral cortex, has six layers, where the properties of layers vary between cortical areas (Brodmann 1909). Individual layers exhibit differing cellular composition and distributions (von Economo and Koskinas 1925), developmental trajectories (Conel 1939), connectivity (Rockland 2015), physiology (Douglas and Martin 1991), and functional roles (Bastos et al. 2012). To date, quantitative measurement of laminar structure has required manual delineation of the layers on histological sections, which is time-consuming, two-dimensional, and largely observer-dependent, with a few exceptions (Schleicher et al. 1999). While whole-brain noninvasive neuroimaging is beginning to resolve laminar-scale features (Yann et al. 2015; Bastiani et al. 2016; Fracasso et al. 2016), the resolution is not yet sufficient to analyze cellular architecture, and MRI signal corresponds more closely to myeloarchitecture than to cytoarchitecture (Schleicher et al. 2005; Glasser and Van Essen 2011). Thus, patterns of cytoarchitecture across the entire brain have not hitherto been characterized. We have therefore developed a fully automated method to identify cortical laminar structures within the BigBrain, a 3D high-resolution histological dataset (Amunts et al. 2013), and to comprehensively quantify classically observed patterns of laminar structure. Such a capability opens new vistas in our understanding of laminar patterns across the brain; moreover, if we can use noninvasive imaging approaches to laminar analyses, it creates the potential for novel insights into structure–function relationships and of characterizing pathophysiology.

Classical histological atlases are the primary source of information on cytoarchitecture, as current noninvasive imaging cannot readily resolve cytoarchitectonic layers. For example, one principle drawn from 2D histological sections was that laminar structure appears to be related to the cortical folds. In particular, the upper cortical layers are thinner at the top of gyral crowns and thicker in sulcal fundi, while lower cortical layers show the inverse relation (von Economo and Koskinas 1925; Bok 1929; Van Essen and Maunsell 1980). Furthermore, both total and laminar cortical thicknesses vary from area to area, in a way that is systematically related to cytoarchitecture, connectivity, and functional specialization (von Economo and Koskinas 1925; Hilgetag and Grant 2010; Wagstyl et al. 2015; Glasser et al. 2016). Drawing on these principles, it has been possible to better localize the laminar origin of structural and functional signals measured in vivo (Muckli et al. 2015; Wagstyl et al. 2016).

In order to fully exploit histological measurements made in 2D, they must be represented in 3D. However, their registration to volumetric MRI is beset with problems. First, measurement is carried out on 2D sections of a 3D curved object, which introduces uncertainties in measurements and errors due to the angle at which the section intersects the cortex. Second, there are also difficulties associated with registering restricted tissue sections back to the entire cortex. Third, manual measurement is time-consuming and highly observer-dependent (von Economo and Koskinas 1925), which places limits on the number and reliability of recorded samples. Together, these manual and two-dimensional limitations have made it difficult, if not impossible, to obtain whole-brain models of the cortical layers. Therefore, while 2D histological studies provide detailed high-resolution insights into regional cortical neuroanatomy, they require extrapolation from a limited number of measurements. There is, therefore, a pressing need for automated methods that measure 3D laminar structure comprehensively.

**Figure 1.**
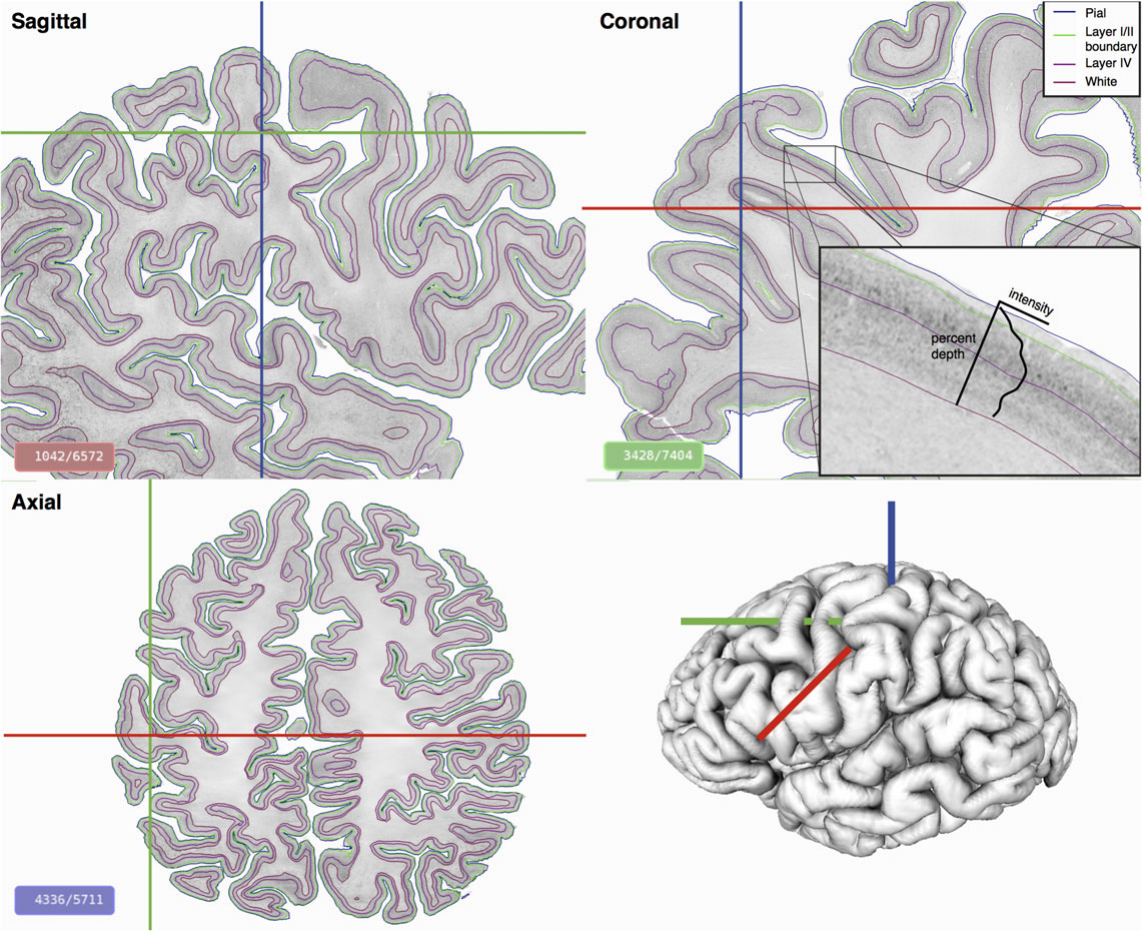
Automatically identified cortical layers on the BigBrain displayed on three orthogonal planes. The brain was sectioned coronally and reconstructed to create a 3D isotropic volume at 20 μm. Cortical intensity profiles (zoomed insert), perpendicular to the cortex, were extracted at all vertices on the surface and used to identify continuous cortical layers. On the gray-scale histological images, minimum intensity pixels are white, maximum are black.

The BigBrain is a unique high-resolution, comprehensive 3D histological model of a complete human brain, including the cerebral cortex (Amunts et al. 2013)(https://bigbrain.loris.ca/). The original 2D coronal sections were stained for cell bodies before being digitized and reconstructed into a 20μm isotropic 3D volume, wherein it is possible to visualize bands of cell bodies corresponding to cortical layers in three orthogonal planes or any oblique angle (Fig. 1) (Borgeat et al. 2007).

We therefore sought to develop a 3D surface-based, automated method for quantitative analysis of laminar cytoarchitecture, based on BigBrain. White and pial cortical surfaces, similar to those used for MRI, had already been reconstructed for the BigBrain (Lewis et al. 2014). Based on these, we first extracted profiles of staining intensity from pial to white surfaces at all vertices. Next, we sought to identify two surfaces related to within-cortical layers—the boundary between layers I and II and a surface within layer IV. The layer I/II boundary was chosen as it is commonly used as the upper bound in analyses of cortical cytoarchitecture (Schleicher et al. 1999, 2005), as layer I exhibits little inter-regional variability. Layer IV was chosen as an important division between supragranular and infragranular layers, which have different connectivities and functional roles (Felleman and Van Essen 1991; Bastos et al. 2012; Markov et al. 2014). Importantly, these layers exhibited relatively consistent features in simulated profiles between cortical regions derived from neuronal density measurements made on 2D sections in the von Economo atlases (von Economo and Koskinas 1925). Critically, these automatically defined surfaces were then verified through comparison with the histology. The layer I/II boundary surface was compared with a manually defined layer I/II and the automatically identified surface within layer IV was tested against manual delineations of the upper and lower boundaries of layer IV. Based on cortical reconstructions, we measured cortical morphology, specifically curvature and layer thicknesses, enabling us to test the relationship between laminar structure and cortical folding across the entire cortex, and mapped the inter-regional variation in the position of cortical layers. Finally, we measured the angles between cortical profiles and the original coronal sections to estimate the measurement errors and limitations for similar 2D analyses. The volumetric and surface data are freely available for download from https://bigbrain.loris.ca/.

## Materials and Methods

### Data Preparation

BigBrain is a 20 × 20 × 20 μm (henceforth described as 20 μm) resolution volumetric reconstruction of the histologically processed post mortem brain, which in full is approximately 250GB in size. Running computations on this amount of data were achieved using a combination of two techniques—subsampling to lower resolutions or dividing the data into manageable blocks. Thus, the data were subsampled at a range of isotropic resolutions 20 μm, 40 μm, 100 μm, 200 μm, 300 μm, and 400 μm. Between 100 μm and 1000 μm, the data could be analyzed as a single volume. For 20 μm and 40 μm, the data were stored into 125 individual blocks, corresponding to five subdivisions in the *x*, *y*, and z directions, with overlap. The overlap of blocks was calculated to be sufficient such that a theoretical cortical column spanning the height of the cortex would be completely contained in a single block. This would enable extraction of complete intensity profiles between pairs of vertices at the edge of blocks without intensity values being altered by boundary effects when the data were smoothed. In order to align intensity distributions with histological and MRI conventions, computations were calculated on inverted images such that background intensity was 0, and image intensity increased with staining intensity. Staining intensity is based on the selective staining of cell bodies and is thus a measure different from the Grey Level Index GLI (Schleicher et al. 1999), which gives an estimate of the volume proportion of stained cell bodies.

### Voxel Resolution

In order to investigate the minimum voxel size required for cortical layer identification, sample profiles were extracted for a vertex in the primary visual cortex (V1) in the calcarine sulcus at isotropic resolutions of 20 μm, 40 μm, 100 μm, 200 μm, 300 μm, 400 μm, and 1000 μm (Fig. S1). The staining intensity of volumes with a voxel resolution between 100 and 1000 μm was sampled from a single image volume, whereas for 20 μm and 40 μm, they were sampled from the overlapping blocks.

### Volumetric Smoothing

Data were smoothed anisotropically using a geometric heat equation and nonlinear smoothing approach (Kimia and Siddiqi 1996). This iterative technique removes small-scale intensity changes, preserving the contours of cortical layers by maximally smoothing in the direction tangential to the cortical layers, to retain interlaminar intensity differences while minimizing the effects of intralaminar intensity variations caused by histological artifacts, for example, circumscribed variations in staining intensities, small defects caused by the sectioning using a microtome (Fig. S2). The overall extent of image smoothing is controlled by the number of iterations, from which the maximum resultant Full Width at Half Maximum (FWHM) of the smoothing kernel can be estimated (Kimia and Siddiqi 1996).

The optimal smoothing kernel was chosen as follows. Profiles were sampled at 40 μm from regions distributed across the four lobes (frontal, occipital, parietal, and temporal lobes). Smoothing was increased until the number of peaks in most profiles was between 3 and 5, approximately the number of peaks expected due to 6–8 cortical layers. These remaining peaks are likely due to interlaminar differences (Figs S2 and S3).

### Tissue Classification

The initial tissue classification was performed on the single-modality cell-body–stained intensities of the individual 20 μm coronal sections. Voxels were classified into two main tissue types (i.e., white matter and cortical gray matter) and background using an artificial neural network trained on manually identified points as priors (Zijdenbos et al. 2002). Over 100 samples were used for each tissue class. The samples were defined manually every 20 sections, then interpolated nonlinearly to the nearby sections. Using a segmentation of the brain for the cerebrum and the cerebellum, the main tissue classes were further differentiated into cortical layer I, a single class for cortical layers II–VI, white matter, subcortical and brainstem gray matter, pineal gland, and cerebellar granular layer (Lewis et al. 2014). Layer I was generally separable as it showed white-matter–like intensities disconnected from white matter.

The data for the 20 μm sections were too large to be reconstructed as a single volume, so the histological and classified sections were assembled into a single volume at 200 μm, which was deemed sufficient for the purpose of cortical gray and white surface extraction. The classified slices at 200 μm were assembled by averaging the tissue fractions of 10 sections at 20 μm, thus improving the signal-to-noise ratio on the 200 μm classified volume.

### Cortical Surface Reconstruction and Registration

The cortical surfaces of the BigBrain were extracted using tools from the CIVET pipeline for in vivo MRI cortical surface analysis (Lepage et al. 2017), adapted for histological volumes (Lewis et al. 2014). High-resolution polygonal mesh surfaces were fitted to the gray–white matter boundary based on the tissue classification of a downsampled 200 μm histological volume. Errors in the placement of the white matter surface, due to factors such as technical artifacts (e.g., remaining tears in the sections and small localized staining artifacts) and tissue misclassifications, were manually corrected on the 200 μm classified volume by overlaying the surface on the 40 μm histological blocks. The white surfaces were then locally adjusted to the maximum intensity gradient of the histological volume.

The white surfaces were subsequently expanded to the gray matter/cerebrospinal fluid (CSF) boundary using the CLASP algorithm (Kim et al. 2005). The resulting white and pial matter surfaces each contain 163,842 vertices per hemisphere, with each pial vertex being linked to its homologous vertex on the white surface. Morphological landmarks—cortical gyri and sulci—were used to register these surfaces to the MRI-based MNI152 average template surface (Lyttelton et al. 2007; Evans et al. 2012). Thus, cytoarchitectural information from the BigBrain can be readily mapped to in vivo neuroimaging data via surface registration.

### Cortical Staining Intensity Profiles

Profiles of staining intensity throughout the cortical depth were created by sampling the BigBrain volumes at 100 equidistant points between linked vertices on the pial matter surface and the white matter surface (Schleicher et al. 1999). Profiles were generated from a 40 μm anisotropically smoothed volume (see Figs S1 and 2 for details on resolution and smoothing parameters).

### von Economo Profiles

**Figure 2.**
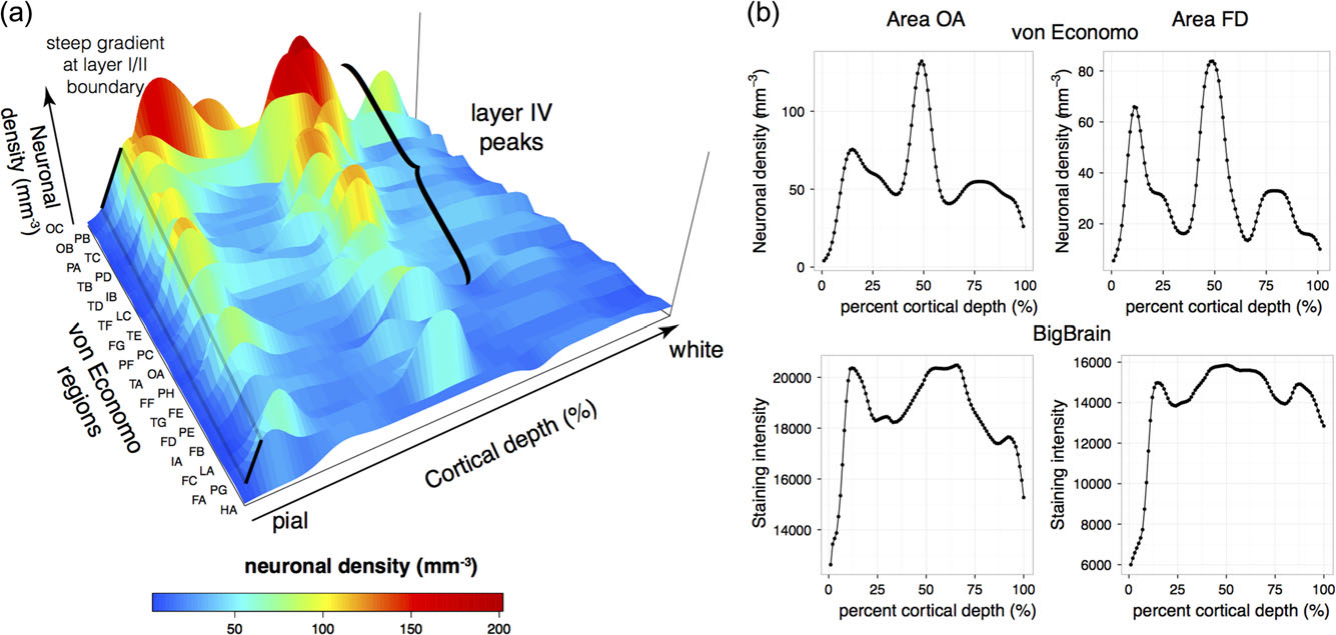
Investigation of density profiles. (*a*) Cellular density profiles were created from areal measurements of thickness and neuronal density of each cortical layer reported by von Economo & Koskinas (von Economo and Koskinas 1925). The first letter of each areal code corresponds to the lobe, the second letter indicates successive lobar measurements e.g., OA, occipital area A, TB, temporal area B, etc. All von Economo profiles exhibited a large positive gradient between layer I, which has the lowest neuronal density, and layer II. Most profiles also exhibited a consistent mid-profile peak corresponding to high neuronal density in layer IV. A notable exception included FA, the agranular motor cortex, so called because no granular layer is visible under a microscope. (*b*) von Economo density profiles were compared with intensity profiles from corresponding areas in the BigBrain. Shown here are von Economo areas OA (extrastriatal occipital cortex) and FD (granular anterior frontal cortex). The layer I/II boundary and mid-cortical layer IV peak is consistently present in both von Economo and BigBrain profiles.

The BigBrain dataset is based on histological sections that were silver stained for neuronal cell bodies (Merker 1983). The cell bodies are more heavily stained, whereas the neuropil remains unstained. Thus, changes in voxel intensity are primarily related to cellular packing density (Wree et al. 1982). To interpret the cortical profiles obtained in BigBrain, we simulated density profiles based on the manual measurements of neuronal density and thickness of each cortical layer in multiple areas carried out by von Economo and Koskinas (Fig. 2*a*) (von Economo and Koskinas 1925). These authors provided numbers for layer thickness and neuronal packing density, which served as a basis to create a histogram-like profile, where the height was given by the density of a layer and the width by the thickness of that layer. For visual comparison, these histograms were then smoothed to the same degree as the smoothed BigBrain intensity profiles. von Economo density profiles were simulated for 30 areas for which measurements were available. Cortical staining intensity profiles were sampled from manually identified corresponding areas in the BigBrain. While there is uncertainty as to the precise location of areal boundaries relative to morphological features (Amunts et al. 2007), we can be more confident of the approximate center of cortical areas. For example, area FA is located in the postcentral gyrus, while PA is at the fundus of the central sulcus. Guided by morphological approximations of the locations sampled by von Economo and Koskinas, we manually identified vertices within each of the corresponding areas on the surface of the BigBrain and extracted the cortical intensity profiles for each (Fig. 2*b*). The von Economo and BigBrain profiles were then visually compared for profile features that were consistent across most cortical areas and which could be used to heuristically identify cortical layers automatically on the BigBrain intensity profiles.

### Identification of Cortical Layers

The cortical intensity profiles extracted between white matter and pial surfaces were used to identify cortical layers. Based on the von Economo profile features, the two most common laminar features were a sharp rise in neuronal density at the boundary between layers I and II, and a peak in neuronal density at the center of layer IV (Fig. 2).

**Figure 3.**
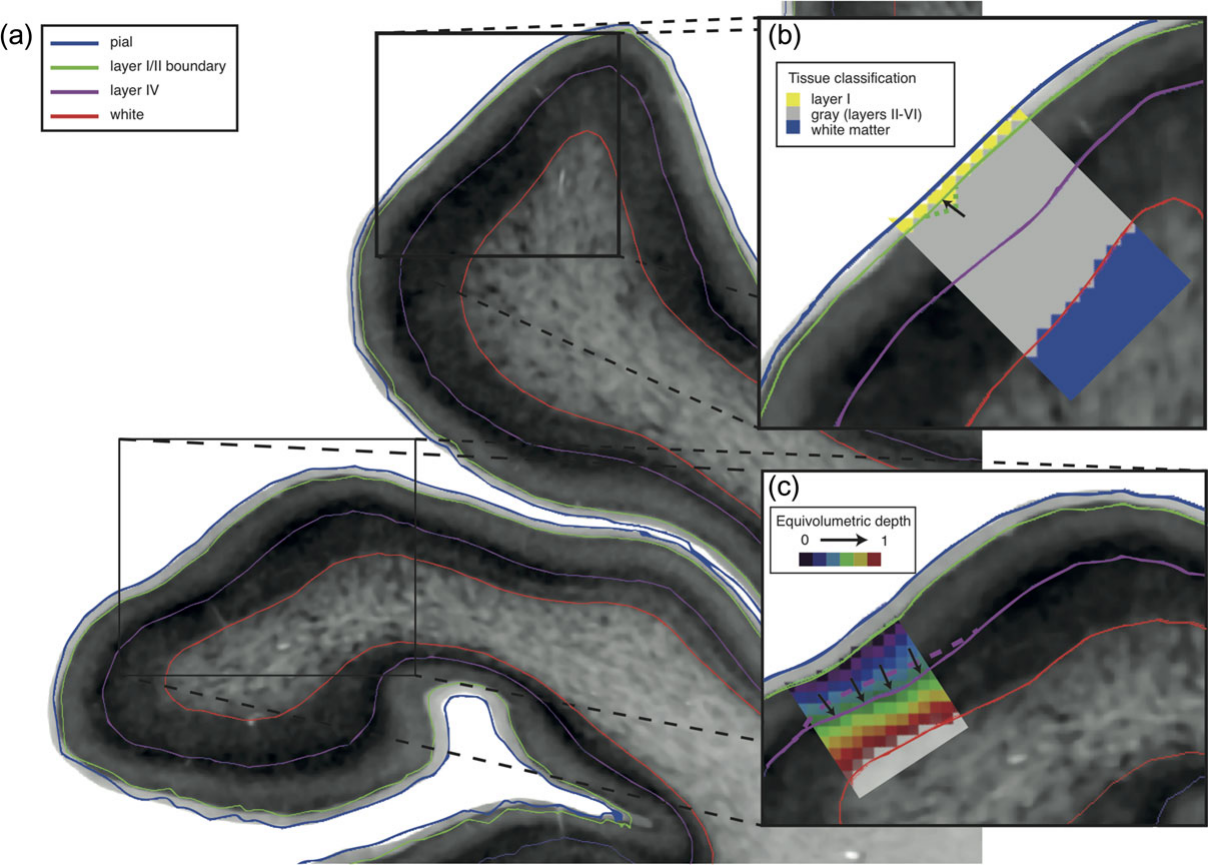
Identification of cortical layers. (*a*) Automatically detected layers from cortical profiles between pial and white surfaces. (*b*) An initial estimate for layer I/II boundary position was placed at the layer I/gray boundary of the tissue classification, made on 200 μm isotropic voxels (yellow/gray voxels). Surfaces were adjusted to the nearest maximum gradient, with two different smoothing steps to produce a smooth surface following the visible boundary between these layers: intensity values were smoothed across the surface and vertices were moved to the position on the profile nearest the smoothed value, and geometric mesh smoothing removed high curvature kinks by averaging neighboring coordinates—as shown by the arrow and the green dotted line. (*c*) The initial position of layer IV was placed at the first major peak after an upwards inflection in the intensity profile, which corresponds to layer II/III. Surfaces were adjusted to the nearest profile maximum, with two iterative smoothing steps: equivolumetric depth values (spectral colored 200 μm voxels) were smoothed across the surface to reposition smooth but inaccurately placed regions (purple dashed line) and geometric mesh smoothing was again used to remove high curvature kinks. On the gray-scale histological images, minimum intensity pixels are white, maximum are black.

The histological profiles (100 points from the 40 μm volume) were intersected with the tissue classification (200 μm isotropic voxels), which served to identify the layer I/II initial boundary. When no appropriately classified voxel was found, the initial estimate was placed at 200 μm below the pial surface (Fig. 3*b*). The position of the layer I/II boundary was subsequently corrected to the nearest inflection point (local maximum gradient in staining intensity). To account for noise artifact and staining inhomogeneities, the surface mesh was improved using the following two steps, iteratively.

- Gradient adjustment: Locally move each vertex to the position of the closest maximum gradient (inflection point) of the staining intensity profile.
- Smoothing on surface: The intensity values at the maximum gradient position for each vertex were smoothed on the cortical surface by three iterations of nearest-neighbor averaging, to remove noise from isolated misplaced vertices. The surface was then adjusted to the point in the profile that matched this smoothed intensity value. When no matching intensity value was found or its position was deeper than the expected thickness of layer I (200 μm), vertices were adjusted to the average depth of their neighbors (Fig. 3*c*).

The mid-layer IV surface was obtained in a similar manner by sampling the histological volumes at 100 points between layer I/II and white surfaces. Layer IV was initially identified as a large peak in intensity, which followed an inflection point corresponding to part of layer II/III (layer II is often but not always a peak; thus, an upward inflection point more reliably marks layer II/III in the profile). This provided an initial estimate for the location of layer IV. To account for noise artifact and staining inhomogeneities, the surface mesh was iteratively improved using the following three steps.

- Geometric smoothing of the mesh without shrinkage (Taubin 1995) (Fig. 3*b*).
- Smoothing of equivolumetric cortical depth (Fig. 3*c*). Equivolumetric cortical depth was calculated for each vertex on a volume at 200 μm. Intensity values that represent the fractional equivolumetric depth between the white and layer I/II surfaces were calculated for each voxel (https://github.com/neurospin/highres-cortex) (Bok 1929; Waehnert et al. 2014; Yann et al. 2015). Equivolumetric depth values were then smoothed across the surface with a 10 mm FWHM isotropic Gaussian kernel (Boucher et al. 2009) and a new surface was created at these depths.
- Local adjustment of each vertex to the nearest peak in the intensity profile for each vertex to generate a new layer IV surface.

The 3 steps were repeated until fewer than 100 of the 163 842 cortical vertices changed location between successive iterations.

### Validation Through Manual Delineation of Histology Sections

To test the accuracy of layer I/II and layer IV surface placement, we compared automatically identified cortical layers against manually delineated cortical layers carried out on a subset of the original 2D histological sections of the BigBrain rescanned at 5 μm resolution, which allows clear identification of single cells. Samples were identified on 7 sections, chosen from those available at 5 μm, to have several suitable portions of cortex in different cortical areas. Sections 1066, 2807, 3300, 3863, 4366, 4892, and 5431 from the total of 7404 were included, representing a range of positions from caudal occipital to rostral frontal. On each of the 7 sections, 6 sample regions were chosen where a number of cortical intensity profiles were within ~5 degrees from the plane of the section, thereby minimizing measurement errors due to oblique cutting planes. For each sample, structures (pial, layer I/II boundary and the upper and lower limits of layer IV, the border between the gray and white matter) were manually delineated. In six of the 42 sample regions, the borders of layer IV were not clearly visible. For two of these, layer IV was reduced (“dysgranular”) to such a degree that a single line was used to label boundary of layers III and V. This was not possible in the final four regions (“agranular”), in which layer IV was not visible. These samples were therefore excluded. A further sample was omitted due to excessive tissue tearing in the original section. The remaining 37 manually delineated regions were then downsampled to 20 μm and registered to the aligned 3D BigBrain volume for comparison with the automatically identified layers.

**Figure 4.**
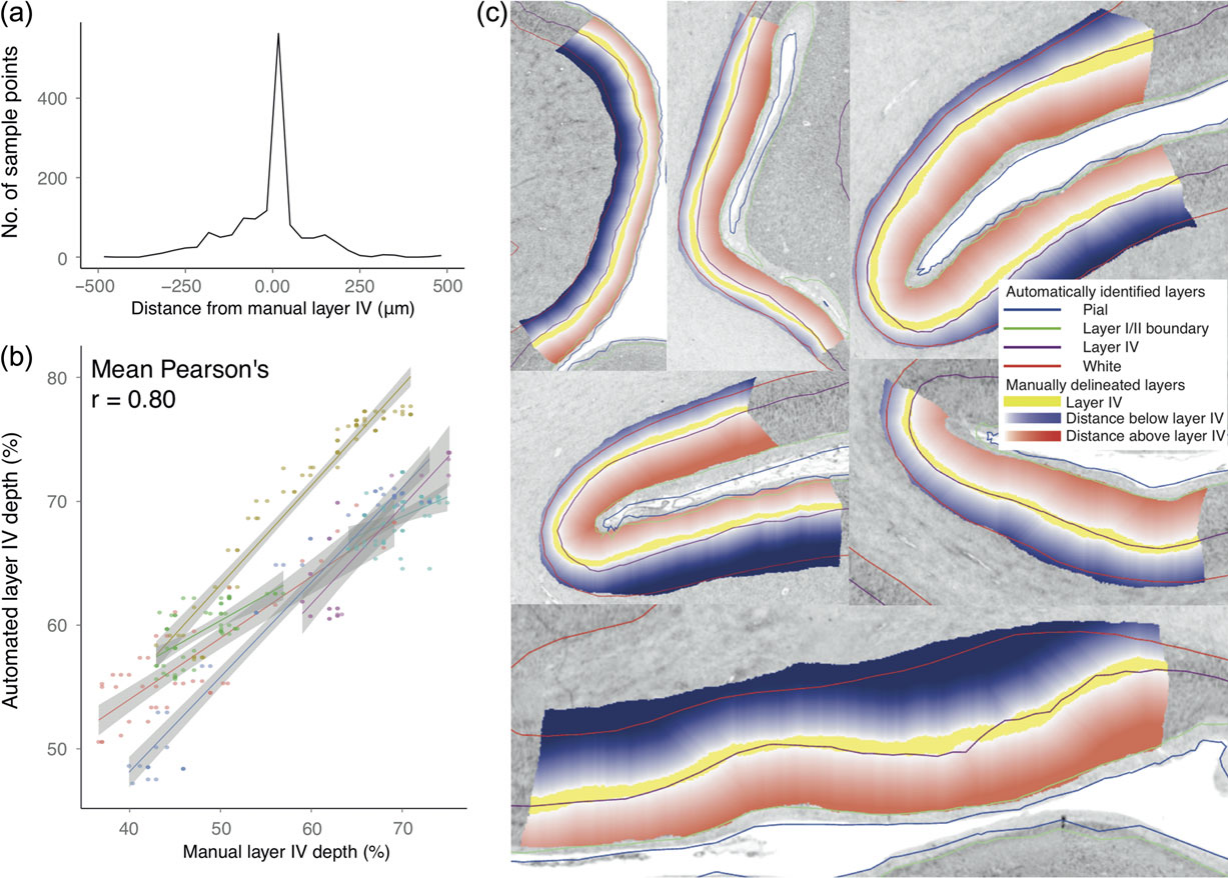
Verification of cortical layers. (*a*) Distance of layer IV vertices from layer IV boundaries manually delineated in 37 areas on 5 μm histological sections. Most points lie directly within or close to layer IV and there is no apparent systematic bias in the small number of points lying outside of these bounds. (*b*) Verification that local, morphologically determined variability in automatically identified layer IV depth followed that of the manually delineated layer IV bounds, shown here for six samples on a single coronal section: 2807. The percentage depth of the midpoint of the manually delineated layers between the white and layer I/II boundary and the percentage depth of the automated layer IV between automated white and layer I/II surfaces was compared for each sample region. The mean Pearson’s correlation coefficient for this section was *r* = 0.80 (across all sections *r* = 0.72). (*c*) Visual validation that layer I/II and layer IV closely follow manually defined boundaries for section 2807 at 20 μm. The automatically identified surfaces have the following colors: white–red, layer IV - burgundy, layer I/II - green, pial–blue. The white–blue overlays indicate distance below the manually defined layer IV/V boundary, with the lower bound at the white surface. The white–red overlays indicate distance above layer III/IV boundary, with the upper bound at the layer I/II boundary. The automatically identified layer IV surface consistently lies within or close to layer IV. On the gray-scale histological images, minimum intensity pixels are white, maximum are black.

In addition to visually comparing the layers, two statistical tests were carried out to verify cortical layer placement. First, absolute distance was calculated between the manually delineated layers and the coordinates at which the two automatically identified surfaces intersect the plane. After testing the distance error for normality with the Anderson–Darling test, a *t*-test was carried out on the mean distance for each sample to test whether the error differed from zero—that is, whether a different surface was being systematically identified. Second, we tested whether within-sample changes in mid-layer IV position matched the variations of layer IV position in the manual annotations (Fig. 4). For the manual segmentations, relative depth of layer IV was calculated by measuring the distance from a line mid-way between the upper and the lower limits of layer IV to the layer I/II layer and white matter surface. Pearson’s correlation coefficient was calculated between relative depth of the automated layer IV and the manually delineated layer IV, for each sample.

### Morphological and Inter-Regional Variations

The relative depth of layer IV at each vertex was correlated against mean cortical curvature to quantify in 3D curvature-dependent changes in the position of layer IV. This relationship was first hypothesized in classical studies of 2D sections (von Economo and Koskinas 1925; Bok 1929). Mean cortical curvature was calculated at each vertex on a mid-surface, which was equidistant between white and layer I/II surfaces. Similarly, the percentage depth of layer IV, taken between the layer I/II and white matter surface, was calculated for each vertex. Both sets of data were smoothed with 3 mm isotropic FWHM Gaussian kernel, to remove isolated extreme values. Surfaces were masked to exclude the medial wall and hippocampal cortex, and vertices where the cortical thickness was smaller than a biologically implausible 0.5 mm—these values generally occurred where the cortex was damaged due to histological processing. Finally, linear, quadratic, and cubic models were used to test the relationship between mean curvature and layer IV relative depth. Bayesian information criteria (BIC) were calculated to compare the model fits (Schwarz 1978). Regional variability in layer IV depth was mapped by calculating the equivalent equivolumetric depth at each vertex.

### Analysis of Cortical Shape

The 3D coordinates of paired pial and white matter vertices were used to calculate the angle at which they intersect the coronal sections and the amount of error this would introduce if measured with 2D cortical histology. The angle α is given by:

$$\arcsin\left(N_{\mathrm{section}}\cdot \;\frac{\left(v_{\mathrm{white}}-v_{\mathrm{pial}}\right)}{\left|v_{\mathrm{white}}-v_{\mathrm{pial}}\right|}\right)$$

where *N* is the normal vector to the section plane and *v*_white_ and *v*_pial_ are the coordinates describing pial and white vertex locations. The overestimation error introduced when measuring the thickness of a piece of cortex where the profile intersects the plane at an angle α is given by:

$$100\;*\left(\frac{1}{\cos\left(\alpha \right)}\;-\;1\right)$$

## Results

### Voxel Resolution

Examining the intensity profiles at a variety of resolutions serves two purposes. First, higher resolution incurs a greater computational cost. Second, it enables us to investigate the minimum resolution required for laminar discrimination. As expected, increasing voxel resolution revealed progressively clearer layer-related variation in cortical intensity (Fig. S1). At 1 mm, clear laminar features were not visible, and layer-related intensity peaks were first visible at around 400 μm. These fluctuations became increasingly well defined as the resolution was increased to 20 μm. Thus, while the highest level of laminar detail was not visible at the limits of resolution for in vivo MRI, certain layer-related features could be detected. However, as resolution was further increased, small-scale fluctuations in intensity also increased, such that at 20μm, there were dozens of peaks in intensity, many within the same cortical layer. Layer-related changes in cortical intensity were sufficient at 40 μm to identify major laminar features, which provided an 8-fold reduction in data volume enabling greater computational efficiency relative to 20 μm volume.

### Anisotropic Smoothing Parameters

To minimize the level of noise, data were anisotropically smoothed, with maximal smoothing in the tangential direction and minimal smoothing across layers in the radial direction (Fig. S2). A range of anisotropic smoothing kernels was tested against number of cortical profiles sampled from across the cortex (Fig. S3). The degree of anisotropic smoothing is determined by the number of iterations, with the effective FWHM being calculated post hoc* (*Kimia and Siddiqi 1996). At 0.163 mm maximum FWHM smoothing, the number of peaks was between 3 and 5. Six-layered isocortex was expected to have 3 peaks (layers II, IV, and VI) and 2 troughs (layers III and V), while additional sublayers such as those found in V1 might give rise to additional peaks. Thus, this range represented an optimal balance between differentiating laminar peaks and minimizing noise-related intensity changes. Subsequent analyses were therefore carried out on 40 μm isotropic voxels, anisotropically smoothed at a maximum FWHM of 0.163 mm (Fig. S2).

### Characteristic Laminar Features of Cortical Profiles

Comparison of profiles from neuronal density changes in von Economo and Koskinas’ neuronal numerical density (number of cells per area) and BigBrain staining intensity profiles revealed characteristic changes (Fig. 2). While there were differences between these profiles due to factors such as nonuniform numerical cell density within layers, variations in cell body size and methods for smoothing data, many features of the profiles were consistent between the two sets of profiles. The most characteristic, consistent feature was a sharp change in intensity at the boundary between layers I and II. This was found in all von Economo profiles and was clearly visible in histological sections. The corresponding feature on the BigBrain profiles was a peak in the first derivative of the profile, close to the pial surface. The second most characteristic feature was a large peak in neuronal density at the center of layer IV, the granular layer. While this peak was not present in all samples, particularly in the “agranular” cortices (e.g., area FA, the primary motor, and premotor areas (Zilles et al. 2015a)), it was present across most other cortical areas (Fig. 2*a*). The corresponding intensity peak was then identified in the BigBrain intensity profiles. Comparison of BigBrain staining intensity profiles with von Economo histological numerical density profiles revealed features that were consistent across most cortical areas and which could therefore be used for automated identification of cortical layers from intensity profiles (Fig. 2).

### Verification of Automatically Identified Cortical Layers

Across the 37 samples, automatically identified layer I/II boundary and layer IV (Fig. 3) closely followed the manually delineated layer bounds (Fig. 4*c*). The absolute distance from the manual delineations was calculated for each vertex within a sample region of interest. These error distances were averaged across the sample to give a mean error for each of the 37 regions. Signed mean distance was calculated to assess for systematic biases in the layers being identified, while unsigned mean distance was calculated to assess the consistency of the accuracy. For signed mean distance, negative distance values indicated that the automated surface was closer to the white matter surface than the manually delineated boundary and positive distance values indicated the surface was closer to the pial surface.

The mean distance between manually and automatically identified layer I/II boundary across all samples was −13 μm (s.d. 40 μm, range −120 μm to 70 μm, unsigned error was 63 μm). To put this distance in context, these surfaces were calculated on 40 μm resolution data. The Anderson–Darling (AD) test indicated that the data were normally distributed (*A* = 0.45, *P* = 0.26) and a one-sided *t*-test indicated that the mean error did not differ significantly from zero (*t* = −1.78, *P* = 0.08). Thus, there was no evidence of a systematic error in which layer I/II was identified. For layer IV, the mean error across all samples was −11 μm (s.d. 68 μm, range −161 μm to +140μm, unsigned error was 72 μm) (Fig. 4*a*). The AD test indicated that the data were normally distributed (*A* = 0.23, *P* = 0.80) and a one-sided *t*-test indicated that the mean error did not differ significantly from zero (*t* = −1.03, *P* = 0.31). Again, there was no evidence of a systematic error—the layer being identified was within layer IV. For each sample, the percentage depth of layer IV between layer I/II boundary and the gray/white boundary correlated strongly with the percentage depth of the middle of the manually delineated layer IV (across all samples mean Pearson’s *r* = 0.72) (Fig. 4*b*). Per-sample measurements of regional accuracy mapped to the cortical surface can be found in Fig. S3. Therefore, the automatically defined layer IV closely followed within-area and between area variations in position of the manually delineated layer IV.

### Layer IV Depth and Cortical Morphology

**Figure 5.**
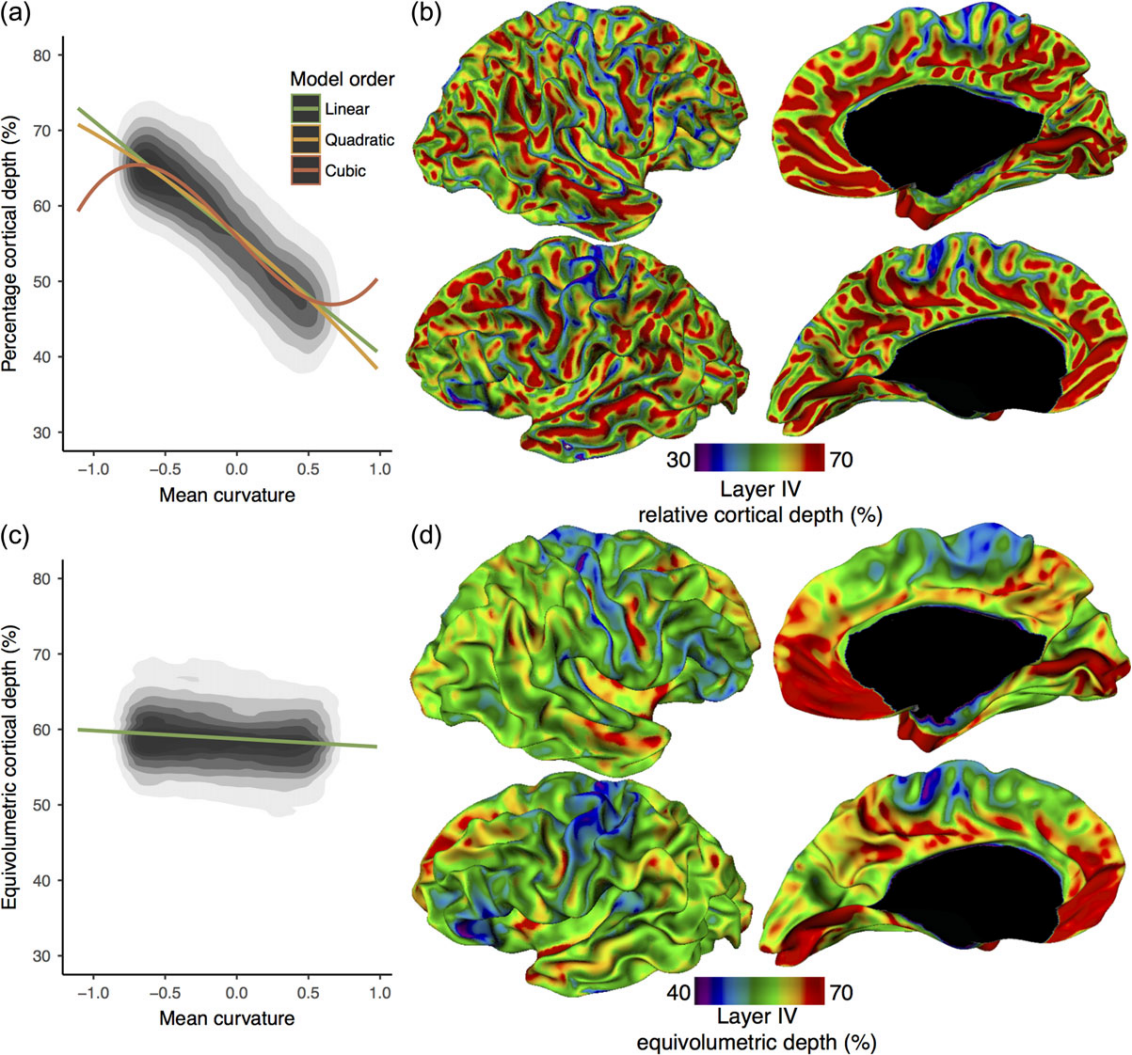
Layer depth and morphology. (*a*) Layer IV percentage depth was strongly predicted by mean curvature (mm^−1^) measured on a mid-surface between layer I/II and white surfaces, being deeper in negatively curved fundi and more superficial in positively curved crowns. The relationship between layer IV depth and curvature was best predicted by a cubic regression. This relationship is consistent with predictions from the equivolumetric model. (*b*) Relative depth of automatically identified layer IV displayed on a spatially smoothed white surface. Consistent with histological studies (Bok 1929), layer IV was located deeper in sulcal fundi and more superficial in gyral crowns. Upper layers (II & III) were therefore relatively thinner in gyral crowns and thicker in sulcal fundi, while the opposite was true for lower cortical layers (V–VI). (*c*) Layer IV equivolumetric depth showed almost no correlation with curvature, lending support to the use of this model in modeling layers in MRI. (*d*) Equivolumetric depth of layer IV on a spatially smoothed white surface. Across cortical areas, there remained a variability in layer IV equivolumetric depth. Layer IV was relatively deeper in the calcarine sulcus (V1) and parts of the medial prefrontal cortex. Due to the presence of border effects and allocortex, areas directly adjacent to the medial wall (black) should be interpreted with caution.

We quantified the relationship between cortical morphology and cortical laminar structure (Fig. 5*a*), a relationship that was first noted in 2D histological studies (von Economo and Koskinas 1925; Bok 1929). Testing across all cortical vertices on both hemispheres, the depth of layer IV was strongly related to mean curvature, as measured on a mid-surface between the layer I/II boundary and white surfaces (Fig. 5*b*). The linear (β=−15.5, F_(1 302273)_ = 4.164 × 10^5^, *P* < 0.0001), quadratic (F_(2 302272)_ = 2.102 × 10^5^, *P* < 0.0001), and cubic (F_(3 302271)_ = 1.483 × 10^5^, *P* < 0.0001) models were all highly predictive. BIC analysis revealed that the cubic model best predicted the relationship (linear: df = 3, BIC = 1893302; quadratic: df = 4, BIC = 1891660; cubic: df = 5, BIC = 1881609). Overall, layer IV was more superficial in gyri and deeper in sulcal fundi. This finding was consistent with classical histological studies including the equivolumetric model of laminar structure and provided 3D evidence that upper cortical layers (I–III) are thinner in gyral crowns but thicker in sulcal fundi while lower layers (V–VI) show the inverse relation. This relationship was present across the entire cortex and, therefore, must be considered when sampling cortical tissue either with histological techniques or for MRI.

There was almost no change in equivolumetric depth with cortical curvature (β=−1.07, *F*_(1 302273)_ = 2706, *P* < 0.0001), suggesting that the model is an appropriate way to account for the relationship between laminar depth and folding. Accounting for the effect of curvature on layer IV depth by using the equivolumetric model of layer depths revealed considerable residual variability position of layer IV depth (Figs 5*c* and 5*d*). Thus, the relative depth of layer IV is determined both by local morphology and by inter-regional differences in cytoarchitecture.

### 3D Histology Overcomes 2D Oblique Sectioning Error

**Figure 6.**
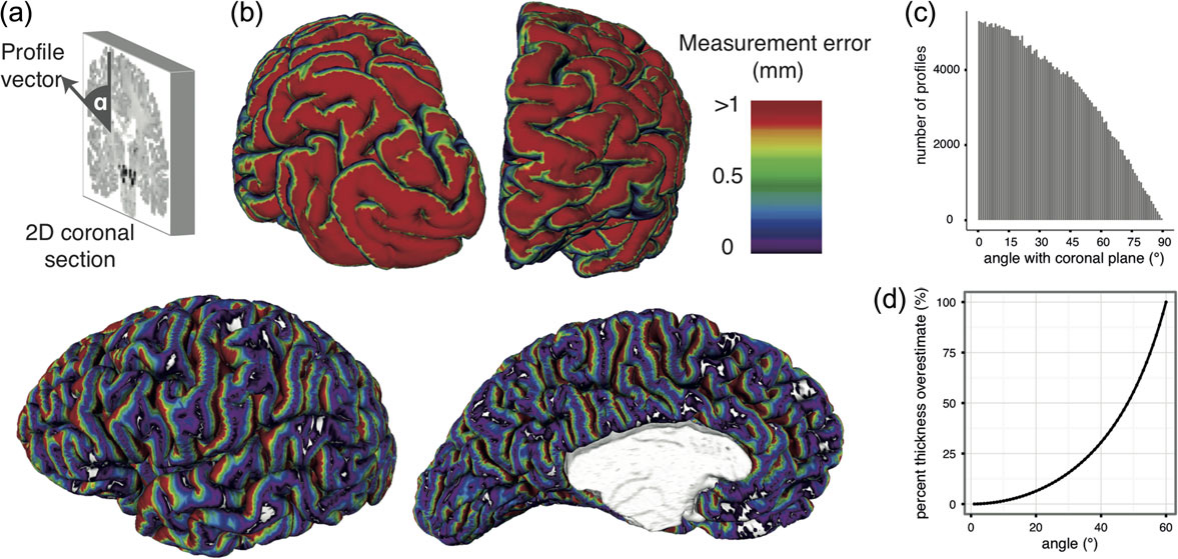
Measurement errors in 2D histology. (*a*) Errors are introduced due to the angle α between the cortical profile vector and the original 2D coronal section. (*b*) Estimated 2D thickness measurement error of a 3 mm cortex due to the angle between the coronal plane and cortical profiles. Red areas indicate errors of 1 mm or greater. Thickness measurements made at vertices colored blue and purple would be little affected by oblique slicing. Occipital (top left) and frontal (top right) views showed many profiles approaching 90° such that a single cortical column would be distributed across hundreds of coronal sections making laminar analysis impossible in 2D. (*c*) Histogram showing the distribution of angles across vertices. At angles above 18°, which made up approximately 69% of vertices, there was a 5% overestimation in any 2D thickness measurement. (*d*) Calculation of the error in thickness measurement associated with a given profile section angle.

Analysis of the angles at which cortical profiles intersected the coronal sections demonstrated how the curved shape of the cortex affects 2D histological measurement (Fig. 6). Importantly, while the 3D BigBrain enables estimation of this error, for most 2D classical histology, these measurements cannot be readily adjusted, as computational reconstruction of the cortical surfaces is required to estimate these angles.

## Discussion

We have developed and validated a fully automated 3D analysis of the laminar structure of the cerebral cortex in the BigBrain. This enabled the identification of two layers across the entire cerebral cortex and revealed cross-cortical patterns of cytoarchitecture. Our results demonstrated that features of cortical staining intensity profiles corresponded to specific cortical layers, and at 40 μm isotropic resolution, these can be used to identify surfaces automatically at the layer I/II boundary and within layer IV (Fig. 1). The accuracy of layer extraction was subsequently corroborated through manual histology. Based on these layers, we have quantified the systematic relationship between cortical layer IV depth and cortical mean curvature and the extent to which the relative depth of this cortical layer changes among cortical areas. These findings have implications for a deeper understanding of cytoarchitectural organization and future directions for in vivo cortical neuroimaging. In particular, they offer exciting possibilities for more precise characterizations of the nature of structural changes associated with neuropsychiatric conditions and for linking such characterizations to functional disturbances.

We used cortical intensity profiles generated to identify histological cortical layers in 3D, at the boundary between layers I and II and a mid-cortical peak in intensity that corresponded to layer IV. The position of the automatically identified layer IV closely varied with the curvature of the cortex (Figs 1 and 5), such that at the top of gyral crowns, it was closer to the pial surface, and in the sulcal fundi, it was closer to the white matter surface, this finding being consistent with known cortical anatomy (von Economo and Koskinas 1925; Bok 1929). These findings further validate the equivolumetric model of laminar structure, which is used to predict the position of layers in MRI, from the curvature of the cortex (Bok 1929; Waehnert et al. 2014). Layer IV depth, as with many other aspects of cytoarchitecture (Welker 1990), varies as much between crowns and fundi within the same cytoarchitectonic area as across cortical areas. Although the precise functional impacts of these differences are unclear, morphology is likely to affect the neurophysiology, connectivity, and therefore perhaps even the functional role of the cortex (Welker 1990; Hilgetag and Barbas 2005). Furthermore, when modeling cortical layers in vivo, it is crucial to account for morphological position to ensure that quantitative sampling is carried out within a single target layer.

After accounting for the close relationship between layer IV position and gyral/sulcal morphology (Fig. 5*a*), there was remaining inter-regional variability in the depths of cortical layers (Fig. 5*c* and 5*d*), which has methodological and neurobiological consequences. Methodologically, neuroimaging studies must consider these regional differences when modeling layer depths in MRI even after accounting for folding. Regional differences in laminar depths mean that a single equivolumetric surface is located in different cortical layers of different cortical areas. Neurobiologically, inter-regional differences in the thicknesses of upper and lower cortical layers support the hypothesis that the cytoarchitecture of different cortical areas is associated with its connectivity (Beul et al. 2017). Variability in layer thickness can arise from a number of linked microstructural properties, including neuronal density (von Economo and Koskinas 1925), dendritic arborization (Elston and Rosa 1998), and myelination (Nieuwenhuys et al. 2015; Zilles et al. 2015b). For example, within the cortical hierarchies, the infragranular layers are thought to be the origin of corticocortical feedback connections, which are relatively sparse in primary sensory regions (Felleman and Van Essen 1991). In the BigBrain, layer IV was very deep (i.e., close to the white surface) in primary visual cortex, which is already thin (Brodmann 1909; Wagstyl et al. 2015). This suggests that layers V and VI are particularly thin in the visual cortex (von Economo and Koskinas 1925), reflecting perhaps the sparsity of cortical feedback connections originating from this area. Further investigation of quantitative cytoarchitecture might help disentangle relationships between laminar thicknesses, intracortical circuitry, and interareal connectivity.

Analysis of cortical profiles between vertices on 3D surfaces enables cytoarchitectural analyses, such as border detection (Schleicher et al. 2005) and quantitative cytoarchitecture (von Economo and Koskinas 1925) across the entire cortex, with several advantages over classical 2D histological techniques. First, 2D histology is limited by the original orientation of the plane of section. 2D histological measurement is restricted to cortical samples cut approximately perpendicularly to the surface. For some obliquely sectioned regions of cortex, measurements can be made, but they are subject to errors introduced by the angle between the section and the surface (Fig. 6); for other areas, the angle is too great and measurement is impossible. Our approach based on the 3D histological reconstructed BigBrain and intensity profiles that can take any oblique angle enabled us to carry out full coverage of the cerebral cortex. Second, 2D manual delineations are labor intensive, even with a limited number of samples. Here, analyses of 325,000 cortical vertices distributed across the entire cortex requires around 40 minutes on a single processor and is entirely reproducible; manual measurement of this many samples is simply not feasible. Moreover, manual measurement is highly observer-dependent—for example, von Economo estimated an interobserver measurement error of up to 500 μm for the placement of the white matter surface (von Economo and Koskinas 1925). For comparison, our automated surfaces had a mean error in automated layer IV position of 11 μm (Fig. 4), with reduced accuracy primarily in regions lacking a clear layer IV (Fig. S4). Therefore, this approach offers several advantages over classical histology in being 3D, rapid, observer independent, and reproducible. These attributes are of great value in exploring larger scale datasets, especially developmental and clinical samples, where structural changes can be subtle.

Finally, these measurements were carried out on a reconstructed cortical surface, complete with the characteristic pattern of gyri and sulci. While cytoarchitectonic and functional areas can be variably related to cortical folds (Rajkowska and Goldman-Rakic 1995; Amunts et al. 1999; Glasser et al. 2016), surface-based landmarks do improve interindividual coregistration and prediction of areal localization (Fischl et al. 2008). Crucially, cortical mesh reconstructions are readily ideally suited for translation between imaging modalities. This has enabled registration of these BigBrain surfaces and their associated cytoarchitectural information to an in vivo average template surface (Lyttelton et al. 2007). Thus, patterns of cortical structure gleaned from these data are transferable to MRI-based cortical reconstructions to generate and directly test novel hypotheses. For example, characterizing gyral–sulcal differences in cytoarchitecture can aid interpretation of morphological change (Wagstyl et al. 2016), and accurate sampling of high-resolution BOLD fMRI signal in specific cortical layers has revealed differential functional responses (Muckli et al. 2015).

A limitation from the analysis of post mortem brains is the effect of tissue shrinkage on measurements. While it is possible to account for this artifact (Amunts et al. 2005), we were primarily interested in the percentage depth of layer IV, on which shrinkage would have little effect (Amunts et al. 1995). Furthermore, there is only a single BigBrain and it is the brain of a single 65-year-old man. Thus, relationships identified here are not necessarily representative of all human brains at all ages. Nevertheless, morphological and inter-regional differences match and extend those reported in classical histological studies, and these insights will aid the future identification of cortical layers in vivo.

This study presents an approach to analyze cortical laminar structure using staining intensity profiles. While the present study was limited to identifying two intracortical surfaces, 1D intensity profiles could potentially be used to further segment the layers and sublayers of the cortex (Figs 2, 3 and S3). However, interareal differences in cytoarchitecture, and by extension profile features corresponding to histologically defined layers, preclude the simple extension of this method (Fig. 3). Instead, future extensions of this approach to fully segment the six layers of the cortex will require more flexible algorithms such as deep learning and could incorporate areal identification alongside laminar segmentation. Importantly, although these techniques were here applied to histological profiles, a similar approach could be used for laminar analysis of cortical profiles derived from MRI (Ferguson et al. 2018).

In summary, we used a fully automated technique to identify cortical layers in 3D on the BigBrain. There was a close relationship between layer IV depth and morphology across the entirety of the cortex, but layer IV depth varied between regions above and beyond this relationship. These patterns can now be used with inter-regional differences to generate strong prior expectations of the location of cortical layers, strengthening our ability to interpret subtle changes in MRI voxel intensity associated with interlaminar differences. Moreover, our analysis characterizing the effects of varying voxel resolution of the BigBrain indicated that high-field MRI is approaching resolutions at which, with suitable sequences, identification of certain cortical layers is feasible. Thus, the principles of laminar structure derived here, and the tools for cortical intensity profile analysis, can be readily used to improve translation between cytoarchitectural studies and in vivo cortical analyses.

## Supplementary Material

Supplementary Data
