# Supplementary figures and images for: Mapping Cortical Laminar Structure in the 3D BigBrain
Source: Cereb Cortex. 2018 Apr 18;28(7):2551–62. doi: 10.1093/cercor/bhy074 (PMC5998962; doi:10.1093/cercor/bhy074)

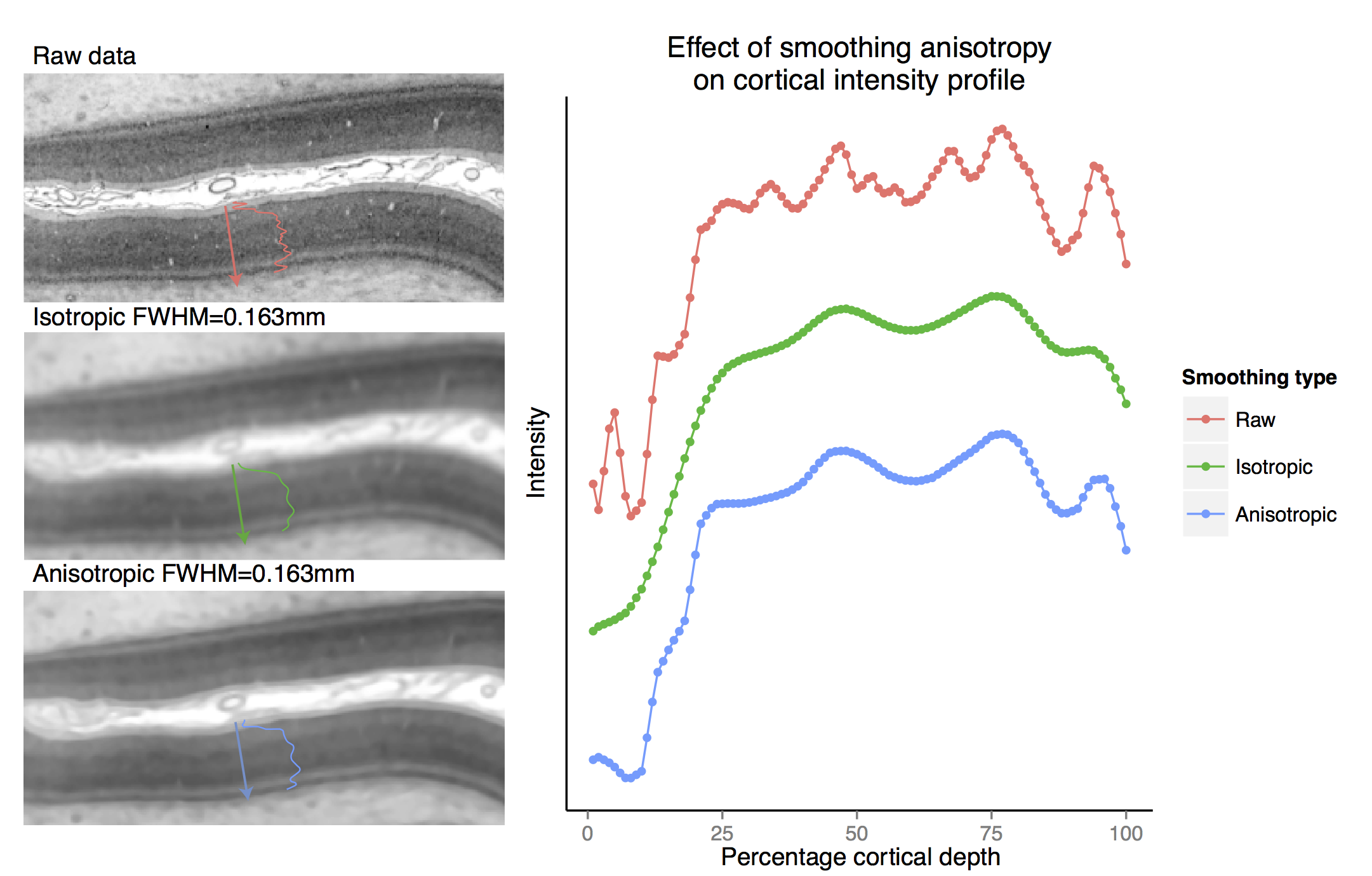

Supplement: Supplementary Data [file bhy074suppl_1.zip › SupplementaryFigure2IsoGeoSmoothing.tiff]

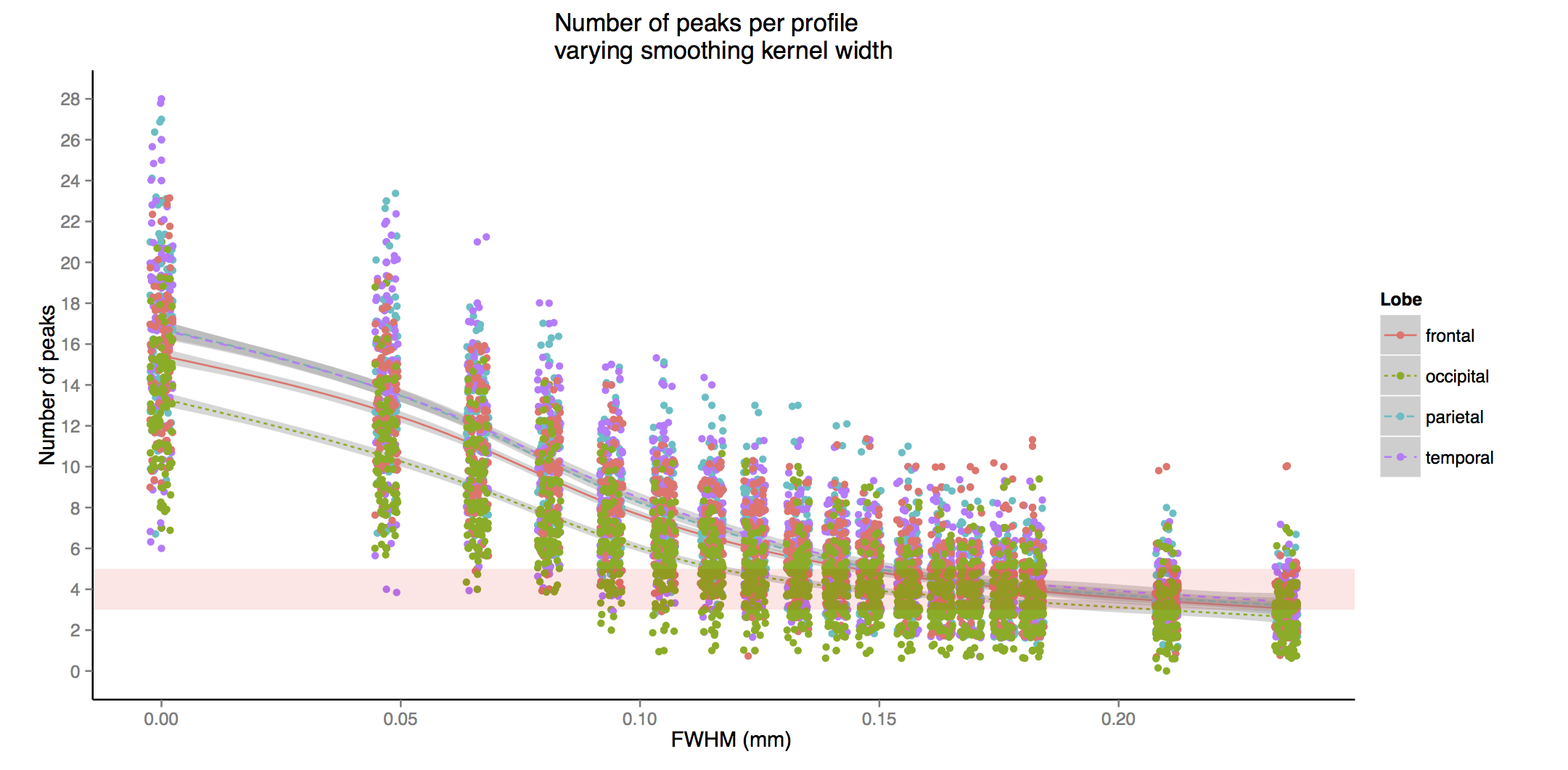

Supplement: Supplementary Data [file bhy074suppl_1.zip › SupplementaryFigure3PeaksPlot.tiff]

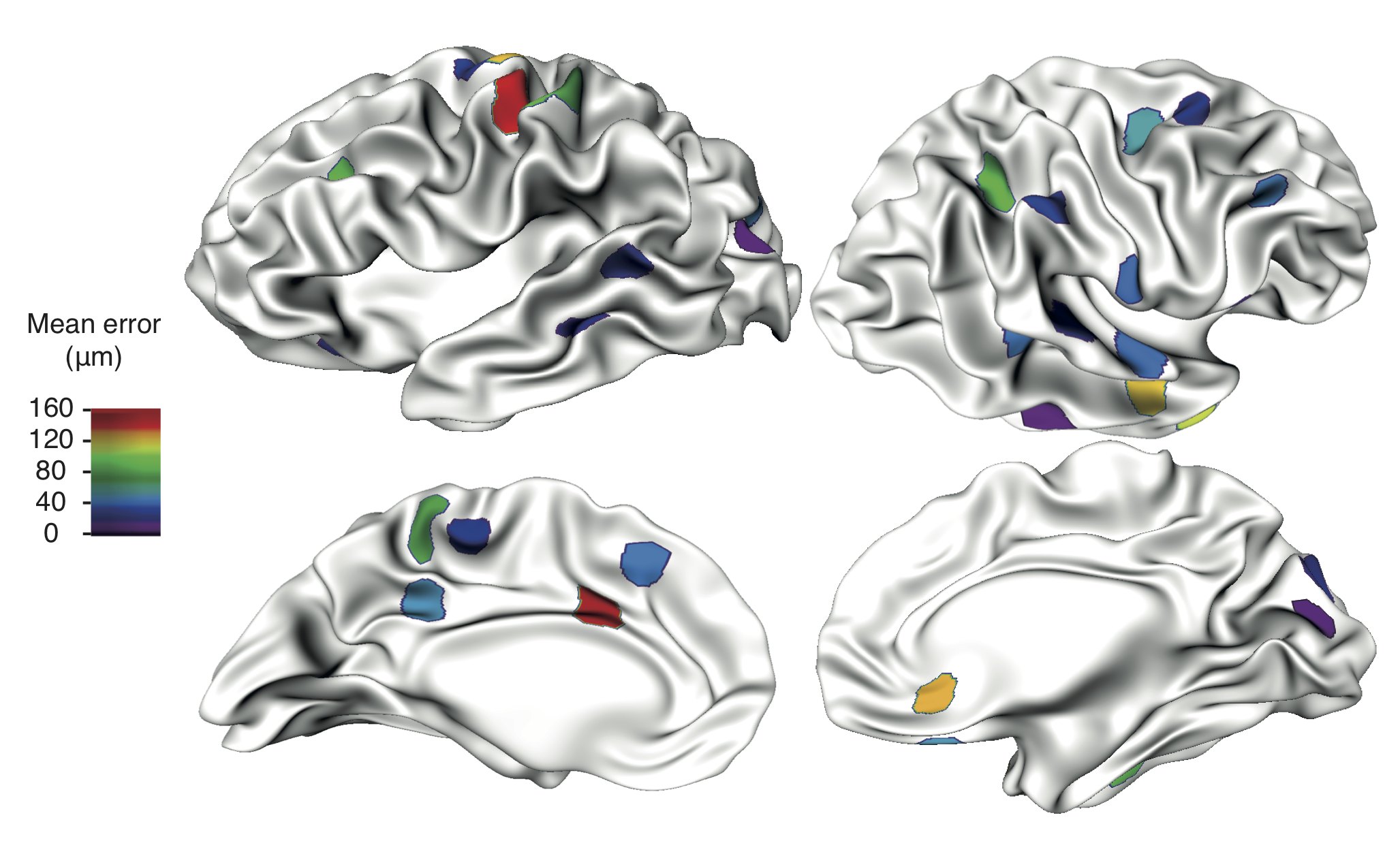

Supplement: Supplementary Data [file bhy074suppl_1.zip › SupplementaryFigure4.tiff]

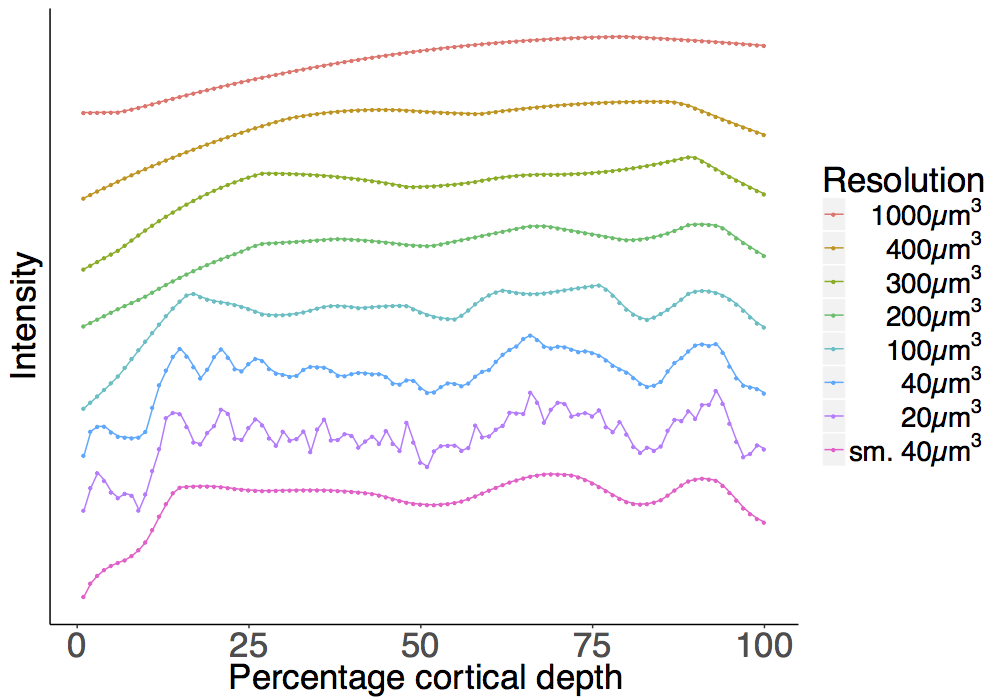

Supplement: Supplementary Data [file bhy074suppl_1.zip › SupplementaryFigure1VoxelResolution.tiff]
